# Monovalent Salt and pH-Induced Gelation of Oxidised Cellulose Nanofibrils and Starch Networks: Combining Rheology and Small-Angle X-ray Scattering

**DOI:** 10.3390/polym13060951

**Published:** 2021-03-19

**Authors:** Kazi M. Zakir Hossain, Vincenzo Calabrese, Marcelo A. da Silva, Saffron J. Bryant, Julien Schmitt, Jennifer H. Ahn-Jarvis, Frederick J. Warren, Yaroslav Z. Khimyak, Janet L. Scott, Karen J. Edler

**Affiliations:** 1 Department of Chemistry, University of Bath, Claverton Down, Bath BA2 7AY, UK; zh603@bath.ac.uk (K.M.Z.H.); VINCENZO.CALABRESE@OIST.JP (V.C.); m.da-silva2@herts.ac.uk (M.A.d.S.); saffron.bryant@rmit.edu.au (S.J.B.); Julien.Schmitt@saint-gobain.com (J.S.); js691@bath.ac.uk (J.L.S.); 2 Food Innovation and Health, Quadram Institute Bioscience, Norwich Research Park, Norwich NR4 7UQ, UK; jennifer.ahn-jarvis@quadram.ac.uk (J.H.A.-J.); fred.warren@quadram.ac.uk (F.J.W.); 3 School of Pharmacy, University of East Anglia, Norwich NR4 7TJ, UK; Y.Khimyak@uea.ac.uk; 4 Centre for Sustainable Chemical Technologies, University of Bath, Claverton Down, Bath BA2 7AY, UK

**Keywords:** cellulose nanofibrils, starch, rheology, SAXS, salt, pH

## Abstract

Water quality parameters such as salt content and various pH environments can alter the stability of gels as well as their rheological properties. Here, we investigated the effect of various concentrations of NaCl and different pH environments on the rheological properties of TEMPO-oxidised cellulose nanofibril (OCNF) and starch-based hydrogels. Addition of NaCl caused an increased stiffness of the OCNF:starch (1:1 wt%) blend gels, where salt played an important role in reducing the repulsive OCNF fibrillar interactions. The rheological properties of these hydrogels were unchanged at pH 5.0 to 9.0. However, at lower pH (4.0), the stiffness and viscosity of the OCNF and OCNF:starch gels appeared to increase due to proton-induced fibrillar interactions. In contrast, at higher pH (11.5), syneresis was observed due to the formation of denser and aggregated gel networks. Interactions as well as aggregation behaviour of these hydrogels were explored via ζ-potential measurements. Furthermore, the nanostructure of the OCNF gels was probed using small-angle X-ray scattering (SAXS), where the SAXS patterns showed an increase of slope in the low-q region with increasing salt concentration arising from aggregation due to the screening of the surface charge of the fibrils.

## 1. Introduction

Cellulose, a polysaccharide, is the most abundant natural polymer [1]. It can be treated to make various forms of nanocellulose such as nanofibrils and nanocrystals to expand its applicability in diverse fields including food, cosmetics, packaging, and pharmaceuticals [2,3]. Their nanoscale dimensions as well as the high density of free hydroxyl groups on their surface make them promising materials for surface functionalisation. For example, 2,2,6,6-tetramethyl-piperidin-1-yl)oxyl (TEMPO) mediated oxidation is commonly used to introduce negatively charged carboxyl groups to the cellulose surface, which is key to enhance the stability of particle dispersions in aqueous media due to electrostatic repulsion forces [4,5]. Cellulose nanofibrils have been extensively used to form hydrogels where the properties can be modulated by various factors such as salts [6,7], pH [8,9], concentration [10,11], temperature [12], and surfactants [13,14,15]. In addition, OCNF hydrogels have been reported to have excellent shear-thinning properties, which is desirable for some formulation-based products, especially in health and personal care applications [13]. However, the gel stability is often affected by the concentration of salt and pH. For example, due to the intense negative surface charge of OCNF (ζ-potential~ −60 mV) [16], the fibrils precipitate in the presence of excess salt due to charge screening [17]. Additionally, stronger gels prepared from a concentrated network precipitate quickly, even in the presence of a smaller quantity of salt due to the aggregation of the highly concentrated particles despite the surface charge, as suggested by McEldrew et al. [18]. Therefore, the addition of filler materials with neutral or lesser surface charge (in the case of particles) or charge density (for polymers) to these highly negatively charged nanofibrils can potentially be utilised to increase their gel strength and at the same time assist in preserving gel stability in the presence of a specific concentration of salt.

Starch belongs to a similar polysaccharide family as cellulose and is also widely used in the food [19] and pharmaceutical [20] industries due to its excellent gelling properties. Starch mainly consists of amylose (linear glucose units) and amylopectin (branched glucose units). The solubility of starch in water mainly depends on the ratio of amylose and amylopectin present in the starch, which depends on their source, such as potato, corn, rice, wheat, maise, and cassava [21]. Additionally, thermo-mechanical treatment is essential to increase the solubility of starch polymers during gelation [22,23]. Starch hydrogels show a tendency to undergo syneresis, leading to a short shelf life [24]. Therefore, the combination of starch with cellulose hydrocolloids has been suggested to improve the stability and rheological properties of starch-based gels [25,26,27]. In addition, cellulose can be utilised as dietary fibre [28], thus blending cellulose derivatives with starches has attracted much interest from food researchers [29].

Rheological measurements have been widely used to study the structure–property relationships between various polysaccharide-based hydrocolloids [27,30,31,32]. For example, viscosities and dynamic moduli of blend gels increased with the addition of carboxymethyl cellulose to waxy rice starch suspension by restricting the molecular mobility within the gel networks formed [27,31]. Xiong et al. investigated the possible interactions between wheat starch and cellulose derivatives such as carboxymethyl cellulose (CMC) and microcrystalline cellulose (MCC) in short-term retrogradation (a process in which gelatinised starch undergoes a disorder-to-order transition) [29]. Being a water-soluble hydrocolloid, CMC chains were suggested to disperse better around amylose during gelation compared to the highly crystalline water-insoluble MCC, as evidenced by rheology.

Both OCNF and starch are anionic hydrocolloids with different charge densities; therefore, understanding the interactions between these two species during gelation is important to describe their rheological properties. The addition of counter-ions (for example, salts) can be utilised to screen the repulsive forces between the OCNF fibrils [6,7] and starch [33]. However, excess counter-ions can lead to complete screening of the repulsive interactions, leading to stronger attractive forces, which often cause gel precipitation and syneresis. ζ-potential measurements can be utilised to investigate surface charge in the presence of external stimuli such as salt and pH [34]. Schmitt et al. recently investigated aqueous suspensions of OCNF at various concentrations and salt concentrations using small-angle X-ray scattering (SAXS) to provide some understanding of the gelation mechanisms [35]. The objective of this study was to investigate the rheological properties of OCNF and starch-based hydrogels as a function of various concentrations of monovalent salt (namely, NaCl) and different pH environments (from 4.0 to 11.5). This provides information on the salt and pH tolerance of these gels when considered as an aqueous rheology modifier in formulation based products. Possible interactions and nanoscale structures were also probed by ζ-potential and SAXS measurements.

## 2. Materials and Methods

### 2.1. Materials

TEMPO oxidised cellulose nanofibrils (OCNF) [4,5] with ~25% degree of oxidation were produced from softwood pulp via a high-pressure homogeniser [36,37]. The OCNF were further purified via dialysis (using cellulose acetate dialysis tubing, MWCO 12400, Sigma-Aldrich, Gillingham, UK) under ultra-pure DI water, 18.2 MΩ cm, for five days (refreshed twice daily). After dialysis, never-dried OCNF suspensions (1 and 2 wt%) were prepared by dilution of the dialysed stock, dispersed using a sonication probe (1 s on 1 s off pulse mode for a net time of 60 min at 30% amplitude, Ultrasonic Processor, FB-505, power 500 W).

Starch (Soluble, S9765) with amylose content ~34% [14] was purchased from Sigma-Aldrich, UK. Starch dispersion (1 and 2 wt%) was also prepared by dissolving the required amount of starch in DI water at 80 °C for 45 min under continuous stirring. Blend gels were prepared by mixing the OCNF (2 wt%) and starch (2 wt%) suspensions (at 1:1 ratio with a total solid content of 2 wt%), followed by vortex mixing until homogeneous hydrogels were obtained. The required amount of sodium chloride (Sigma-Aldrich, UK) was added to the starch solution prior to mixing with OCNF. The pH of the gels was adjusted using HCl and NaOH solutions. Ultra-pure DI water (18.2 MΩ cm) was used for dilutions and sample preparation.

### 2.2. Characterisation

#### 2.2.1. Transmission Electron Microscopy (TEM)

Transmission electron microscopy (TEM, JEM-2100 Plus, JEOL Ltd, Peabody, MA, USA) analysis was carried out to investigate the morphology of the OCNF at an operating voltage of 200 kV. A dilute OCNF suspension (0.025 wt%) was added on a Cu-grid (mesh size 300), then negatively stained using uranyl acetate (from Sigma-Aldrich, Gillingham, UK) (2 wt%) for enhanced contrast during TEM measurements [14].

#### 2.2.2. High-Performance Liquid Chromatography-Size exclusion Chromatography (HPLC-SEC)

Starch samples (4.00 mg) were dissolved in 1.0 mL DMSO (99.5% pure; Sigma-Aldrich, UK) with 0.5% (*w*/*w*) LiBr (Sigma-Aldrich, UK) while pullulan standards (PSS-pulkit, Polymer Standard Service, Mainz, DE) were prepared at 2 mg mL^−1^ and incubated at 80 °C in glass vials overnight. A Perkin-Elmer (Llantrisant, UK) Series 200 High-Performance Liquid Chromatography (HPLC) equipped with a refractive index detector (RI), autosampler (35 °C), and column heater (80 °C) was used for peak resolution by size exclusion. An isocratic mobile phase of DMSO with 0.5% LiBr (*w*/*w*) at a flow rate of 0.500 mL min^−1^ and a stationary phase involving a series of columns beginning with a guard column (8 × 50 mm, GRAM; Polymer Standard Service, Mainz, DE) and two analytical columns in series (10 μm; 300 Å followed by 30 Å; 8 × 300 mm, GRAM; Polymer Standard Service, Mainz, DE). Total run time was 60 min, and injection volume for each sample was 50 μL, calibration curves were generated using pullulan standards with peak molecular weights ranging from 342 to 708,000 Da and correlation coefficients of R^2^ = 0.9996 ± 0.0001 (Figure S1), similar to methods described by Cave et al. [38]. The relationship between elution volume and hydrodynamic radius (*R*_h_) was determined using Mark–Houwink parameters for pullulan in DMSO/LiBr solution at 80 °C with the following parameters: K = 2.424 × 10^−4^ dL g^−1^, α = 0.68, and the dn/dc value was 0.0853 mL g^−1^ [39].

#### 2.2.3. Rheological Analysis

A stress-controlled rheometer (Discovery HR-3, TA Instruments, USA) equipped with a sandblasted plate-plate stainless steel geometry (40 mm) was used to perform the rheological tests at 25 °C, as previously described [15]. Gels (approximately 0.7 mL) were placed in between the plates and frequency, and amplitude sweeps and flow curves were measured (with a plate-plate gap of 0.5 mm). A thin layer of low viscosity silicone-oil was added to the edge of the geometry to prevent evaporation. Frequency sweeps were conducted within the linear viscoelastic range in strain control mode at 0.5% strain with an angular frequency range from 0.1 to 100 rad s^−1^. Amplitude sweeps were measured at an angular frequency of 1 Hz (6.28 rad s^−1^), covering the strain ranging from 0.5 to 50%. Finally, flow curves were measured to study the viscosity response of the sample to shearing, with a shear rate ranging from 0.01 to 100 s^−1^.

#### 2.2.4. Surface Charge

Dilute suspensions (10 times dilution) of samples, using the appropriate buffer solution (DI water, NaCl, or pH adjusted buffer) were used to measure the apparent ζ-potential utilising a Malvern Zeta-sizer Nano ZSP^®^ (Malvern, UK) [40]. Samples were placed in the folded capillary electrode cell and were equilibrated at 25 °C for 120 s prior to testing. The data were taken from an average of five measurements from 100 scans each.

#### 2.2.5. Small-Angle X-Ray Scattering (SAXS) Analysis

SAXS measurements were conducted at Diamond Light Source (Didcot, Oxfordshire, UK), on the I22 beamline [16,35] (using an X-ray wavelength of 1 Å corresponding to an energy of *E* = 12.4 keV) equipped with a PILATUS P3-2M detector (Silicon hybrid pixel detector, DECTRIS). Samples were loaded in glass capillary tubes (diameter~1.5 mm) and sealed. An empty capillary and the capillary containing DI water were also recorded for solvent subtraction [35]. The probed *q*-range was 0.005–0.2 Å^−1^, where *q* is the scattering vector.

SASView software (version 4.1.2) was used to fit the SAXS data using either a rigid and flexible elliptical cylindrical model [35].

The intensity *I(q)* can be written as follows:

(1) I(q)∝ P(q) S(q)

with *P(q)* the form factor of the objects studied, giving information about their shape and *S(q)* is the structure factor associated with the interactions between the objects probed.

The structure factor, *S(q)*, was obtained by dividing the pattern obtained in the presence of salt by the pattern from OCNF without salt, where fibril–fibril interactions could be neglected:

(2) $$S\left(q\right)=\;\frac{I\left(q\right)}{k.I_{no\;salt}\left(q\right)}$$

where *S(q)* is the structure factor of the gel prepared in the presence of salt (NaCl = 0.25, 0.5, 0.75, or 1 wt%); *I(q)* is the scattered intensity of the same gel; *I_no salt_(q)* is the intensity of the OCNF gel without salt and with *k* a factor adjusted for each sample to have the same intensity at large angles where no structuring is seen.

## 3. Results and Discussion

**Figure 1:**
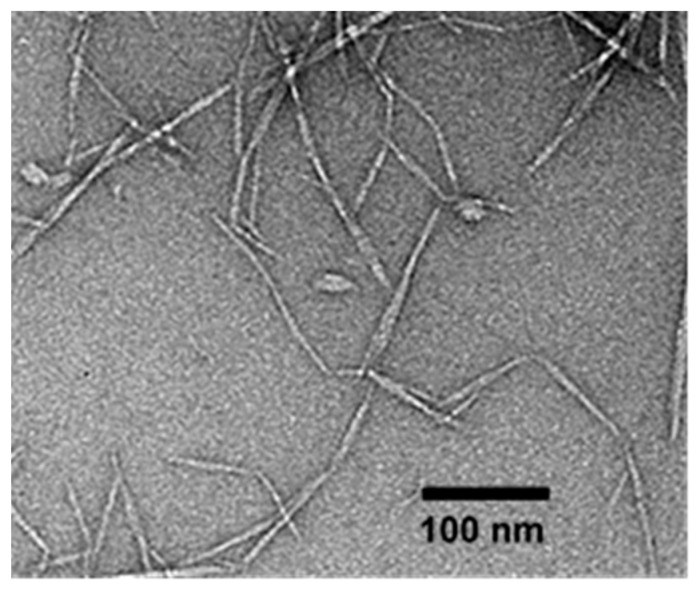
Transmission electron microscopy (TEM) image of never-dried oxidised cellulose nanofibrils (OCNF).

The morphology of the OCNF, as characterised via TEM (see Figure 1), revealed a long fibrillar structure with a diameter of 7 (±2) nm and a length of 160 (±60) nm (from averaging 175 measurements, reported previously) [35]. The ζ-potential value of the OCNF was found to be −56 (±2) mV.

**Figure 2:**
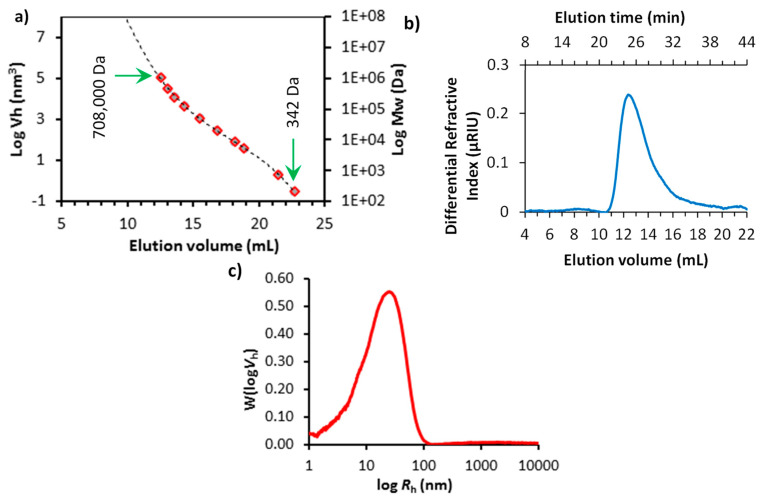
(**a**) Standard curve obtained from the elution profiles of pullulan standards by size exclusion (SEC) HPLC, presented as log(hydrodynamic volume, *V*_h_) vs. elution volume (molecular weights of the pullulan standards are also presented on the right-hand *Y*-axis). (**b**) Elution profile of averaged starch samples (n = 2) and (**c**) average chain length distribution of starch: SEC weight distribution, W(log*V*_h_), expressed as a function of hydrodynamic radius (*R*_h_) of pullulan standards (the elution profiles of the pullulan standards can be seen in Figure S1).

The size distribution of the soluble starch was characterised by the HPLC-SEC method using pullulan standards. Figure 2a shows the calibration curves of pullulan standards with peak molecular weights ranging from 342 to 708,000 Da (please see Figure S1 for the elution profiles of the pullulan standards). Cave et al. suggested that the elution volume could be related to the associated hydrodynamic volume, allowing calculation of the hydrodynamic radius of a sample over the size range of these standards [38]. The elution profile of the starch sample is presented in Figure 2b, and the average chain length distribution of the starch sample as a function of hydrodynamic radius (*R*_h_) of the pullulan standards are shown in Figure 2c. The true size distribution of a starch sample using a chromatographic technique is challenging to derive [41]. Starch is composed of linear amylose and highly branched amylopectin components, and the amylopectin fractions, in particular, are thought to undergo significant chain scission during the solvent and temperature assisted separation technique involved in SEC [38,42]. Therefore, in this study, the *R*_h_ of the starch sample was considered to give an approximate size distribution for this material. The peak *R*_h_ of the starch was found to be around 31.3 nm (calculated from Figure 2c using Mark–Houwink parameters for pullulan standards [39]). Thus, the size distribution of the dissolved starch species was about five times smaller than the length of the OCNF fibrils.

**Figure 3:**
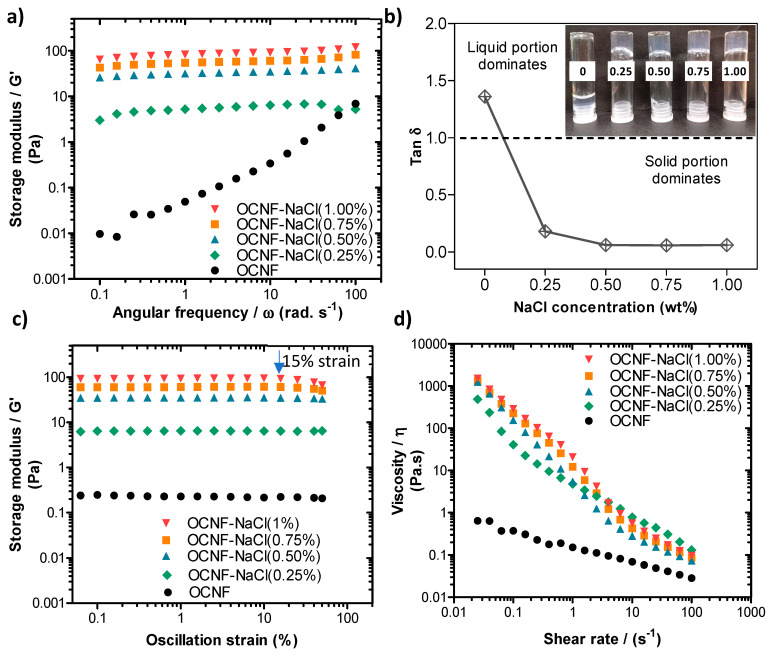
(**a**) Oscillatory frequency sweeps, (**b**) tan δ values obtained at a frequency of 1 Hz (6.28 rad s^−1^), (**c**) oscillatory amplitude sweeps, and (**d**) shear flow curves of OCNF (1 wt%)/NaCl (0, 0.25, 0.5, 0.75, or 1 wt%) hydrogels. Inset in (**b**) is a photograph of the different samples, labelled by their salt concentration (in wt%). The blue arrow in (**c**) indicates the oscillation strain at which the sample at 1 wt% NaCl deviates from the linear viscoelastic (LVE) regime.

The addition of NaCl salt at various concentrations (0.25, 0.50, 0.75, and 1.00 wt%) to the aqueous dispersion of OCNF (1 wt%) was studied to determine the effect on the rheological properties. Fukuzumi et al. reported that TEMPO-oxidised cellulose nanofibrils in dilute aqueous dispersions (0.1 wt%) were homogeneous up to 50 mM NaCl (~0.29 wt%) and formed aggregated gel particles at 100 mM NaCl (~0.58 wt%), but the gel became separated into two phases (gel and supernatant) at ≥200 mM NaCl (~1.16 wt%) [6]. Therefore, mixtures of OCNF (1 wt%) with >1 wt% NaCl were avoided in this study. The storage moduli (G′) of OCNF gels were seen to increase with the salt content, as illustrated in Figure 3a. The pure OCNF gels (1 wt%, without salt) had a frequency-dependent linear increase of G′, suggested to arise due to dynamic transient interactions among the fibrils [43]. Addition of salt to the OCNF (1 wt%) dispersion caused the mixture to show a less frequency dependent G′, represented by the negligible slope of the curves in Figure 3a. Increasing ionic strength screens electrostatic repulsion forces between the cellulose fibrils, hence enhancing fibril–fibril attraction, resulting in a denser fibrillar network and the formation of a stiffer gel [35]. This is in agreement with the tan δ (=G″/G′, with G′ and G″ being the storage and loss modulus, respectively) curves obtained for the OCNF/salt gel system at a fixed frequency of 1 Hz (6.28 rad s^−1^) (as presented in Figure 3b). The addition of salt to the OCNF suspension results in drastic changes in tan δ, which goes from >1 (i.e., G″ > G′) characteristic of fluid in the absence of salt to tan δ < 1, which is the signature of a gel, after salt addition. The photos (inset in Figure 3b) also demonstrate the formation of self-standing stiffer gels in the presence of salt. Here, the addition of 0.50 wt% (~86 mM) NaCl content to the OCNF gel (1 wt%) was sufficient to achieve stronger interactions among the fibrils without any noticeable precipitation, as at higher salt concentration, no significant change in the tan δ values were observed.

From the amplitude sweep curves (Figure 3c), the linear viscoelastic (LVE) region of all the OCNF/salt gels, except for the gels with 1 wt% NaCl (~171 mM), were found to extend to a strain of 40.0%. This demonstrated that there was no loss of the quiescent gel structure due to the applied strain. However, the OCNF gels containing 1 wt% NaCl reached the end of the LVE region and started collapsing at 15% strain (blue arrow in Figure 3c) due to the breakage of the aggregated gel network at this deformation.

Figure 3d shows the flow behaviour of the gels, where the dependence of apparent viscosity (*η*) with respect to the shear rate of the gel systems are presented. The OCNF (1 wt%) gels with various concentrations of NaCl possessed shear thinning properties. The addition of salt to the OCNF gels (0.25 wt% NaCl) induced a significant increase of *η* and shear dependency.

Starch suspensions (1 wt%) with various salt contents were also trialled for gel formation. In the presence of salt, the starch dispersions were weakly viscous liquids (see Figure S2). The storage modulus of this type of flowing and liquid-like dispersion is difficult to measure in oscillatory mode due to the absence of any entangled gel network. Therefore, the G′ values obtained for various concentrations of starch/salt dispersions (Figure S2) are mostly associated with instrumental noise, and the upturn observed at a higher frequency is an artefact due to inertia. Gelation of starch decreased in the presence of NaCl. This was due to the fact that Na^+^ ions compete with the starch for the bound water in the gel system, resulting in less retrogradation, as suggested in the literature [44,45,46,47].

**Figure 4:**
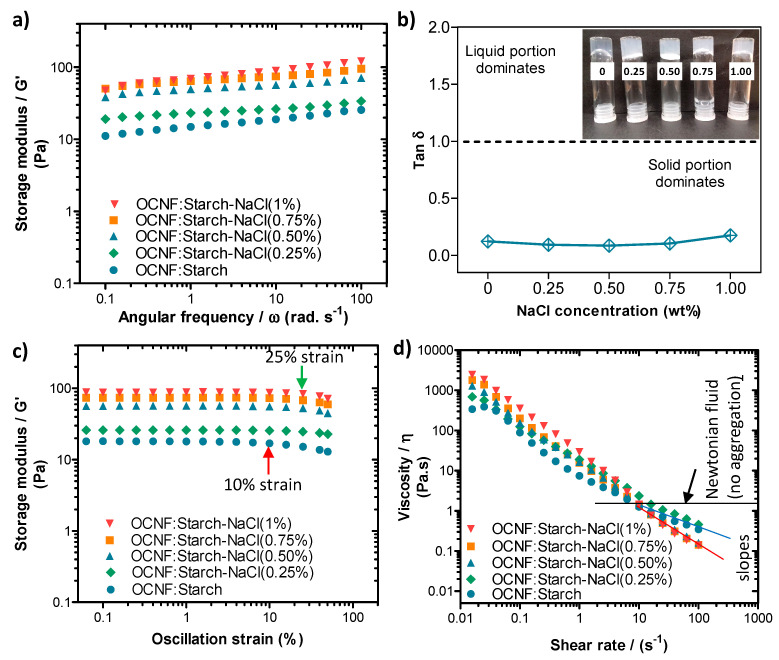
(**a**) Oscillatory frequency sweeps, (**b**) tan δ values obtained at a frequency of 1 Hz (6.28 rad s^−1^), (**c**) amplitude sweeps, and (**d**) shear flow curves of OCNF:starch (1:1 wt%)/NaCl (0, 0.25, 0.5, 0.75, and 1 wt%) hydrogels. Inset in (**b**) is a photograph of the different samples, labelled by their salt concentration (in wt%).

The blended hydrogels were prepared by mixing an OCNF dispersion with a starch suspension at a ratio of 1:1 wt% and various concentrations of NaCl. Their rheological properties are presented in Figure 4. Here, the OCNF:starch (1:1 wt%) gels in the presence of salt showed low-frequency dependent G′ values (Figure 4a). Similar to the OCNF/salt gels, OCNF:starch/salt gel systems also revealed the greatest increase in their interactions at 0.50 wt% NaCl content. Above this concentration, the further improvement in their rheological properties was not remarkable. It is to be noted that OCNF:starch (1:1 wt%) (without any salt) formed solid-like gels (tan δ < 1), as can be seen in Figure 4b. This might be due to the presence of a higher weight fraction of solids (2 wt% in total) as well as their contribution toward the formation of entangled networks. Although the pure starch gels containing similar solids content (i.e., 2 wt%) revealed significantly lower values of G′ compared to the OCNF:starch (1:1 wt%) gels, the pure OCNF (2 wt%) showed slightly higher G′ compared to the OCNF:starch (1:1 wt%) gels [14]. This was expected as the OCNF are the main building block for stiffer gel formation in these systems due to their fibrillar structure (higher aspect ratio, Figure 1) as well as their higher negative surface charge (ζ-potential~ −56 mV) [16]. Therefore, we suggest that no synergistic effect was observed for the OCNF:starch (1:1 wt%) gels and the properties of these gels are simply additive.

From the amplitude sweep curves (see Figure 4c), all the OCNF:starch (1:1 wt%) gels showed the LVE region extended up to at least 10.0% strain (indicated by the red arrow) reaching even 50.0% strain when 0.25 wt% NaCl was added. This suggests that the salt-induced aggregated gel network can support greater deformation before breakage, possibly due to the augmented interactions between the building blocks of the gel. Further addition of salt content to the OCNF:starch gels showed a reduction of the LVE region to 25% strain (indicated by the green arrow in Figure 4c). After the LVE region, the slightly downshifted curves suggested the initiation of collapse of the larger aggregated gel networks at higher strain.

The OCNF:starch gels in the presence of various concentrations of NaCl also showed similar shear-thinning properties, as can be seen in Figure 4d. A small increase in the shear viscosity (*η*) of the OCNF:starch gels were observed with the addition of salt. However, after a 10 s^-1^ shear rate, a downshift in slope for higher salt content OCNF:starch gels (0.50 wt% salt and more, as indicated by the solid red line in Figure 4d) was observed compared to the slope of OCNF:starch gels without salt or with 0.25 wt% salt (demonstrated by the solid blue line in Figure 4d). This also suggests a significant level of aggregation in the gel networks, which correlated well with the amplitude sweep curves obtained for the same gel system.

**Figure 5:**
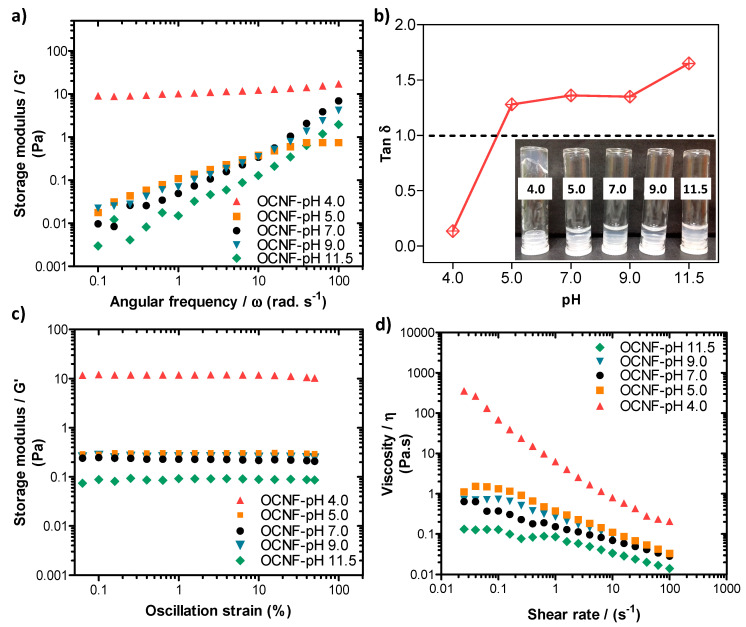
(**a**) Oscillatory frequency sweeps, (**b**) tan δ values, (**c**) amplitude sweeps, and (**d**) shear flow curves of OCNF (1 wt%) dispersions at various pH conditions (pH 4, 5, 7, 9, and 11.5). Inset in (b) is a photograph of the samples labelled by their pH.

Rheological properties of the pure OCNF (1 wt%) dispersions at various pH conditions were also measured, and the data are presented in Figure 5. The pH was adjusted (ranging from 4.0 to 11.5) by adding 10–30 µL of various concentrations of HCl and NaOH solutions to the 5 mL dispersions (here, the change in overall wt% due to the addition of acid or base was neglected). The OCNF (1 wt%) dispersions showed similar frequency dependant G′ curves at pH 5.0–9.0, demonstrating their stability over this range (Figure 5a). Tan δ values of these gels obtained at pH 5.0–9.0 were also found to be similar (tan δ > 1, presented in Figure 5b) as well as the dominant viscous behaviour (inset of Figure 5b). However, at pH 11.5, the OCNF (1 wt%) gels showed precipitation as indicated by an even higher tan δ value as well as lower G′ values compared to the control OCNF gels obtained at pH 7.0. This could be due to the role of Na^+^ and OH^-^ ions (which were used to increase the pH) in the OCNF gel system, similar to the effect of salt, where Na^+^ ions are assumed to screen the electrostatic repulsion between fibrils. In contrast, the G′ value of the OCNF gels obtained at pH 4.0 was found to be significantly higher as well as less frequency-dependent (represented by the negligible slope of the curves in Figure 5a) due to the protonation of carboxylic groups at the surface of OCNF, resulting in a moderate screening of the electrostatic repulsion forces and leading to stronger inter-fibrillar interactions. The tan δ value (tan δ < 1) and the photographs (see inset Figure 5b) also indicate the formation of a stronger gel at pH 4.0. Additionally, in this study, much stiffer OCNF gels were produced at pH 3.4. This was due to a higher degree of protonation of COOH groups, and a significant reduction in their electrostatic repulsion forces, leading to the formation of more aggregated networks. As a result, clear phase separation was observed for the OCNF (1 wt%) gels prepared at pH 3.4 during rheological measurement (Figure S3). A self-standing gel at low pH (<3.0) and free-flowing dispersion high pH (11.0) of TEMPO-oxidised cellulose nanocrystals (1 wt%) were similarly reported to form by Way et al. [48].

From the amplitude sweep curves (see Figure 5c), all the OCNF gels prepared at pH 5.0–9.0 showed similar G′ values, while G′ of the same gel at pH 4.0 was found to be significantly higher. Additionally, the LVE region of the gel at pH 4.0 was seen to be extended to 50% strain, suggesting the stability of this gel at higher amplitude (i.e., no breakdown of the gels after applied strain), which is in good agreement with the photographs of the gels obtained after rheology measurements (see Figure S3). Similar trends were also observed for the viscosity curves of these pH-induced OCNF gel systems (presented in Figure 5d), except for the dispersion obtained at pH 9.0, where a slight increase of viscosity was observed up to a 1 s^−1^ shear rate compared to pH 7.0. This could be due to the role of Na^+^ ions (from NaOH) added to the gel system to achieve pH 9.0. However, a higher amount of Na^+^ ions present in the pH 11.5 gel system caused significant aggregation and phase separation, hence comparatively, a lower viscosity value was obtained, as discussed earlier.

**Figure 6:**
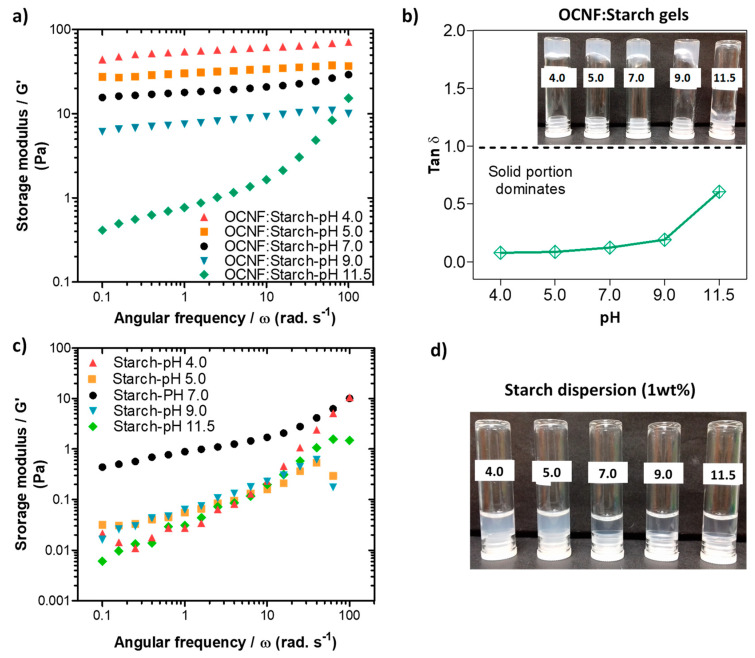
(**a**) Oscillatory frequency sweeps and (**b**) tan δ data for the OCNF:starch (1:1 wt%) blends at different pH (4, 5, 7, 9, and 11.5). Inset in (b) is a photograph of the samples labelled by their pH. (**c**) Frequency sweep curves and (**d**) photograph of the control starch (1 wt%) dispersions produced at the various pH values.

In the presence of starch, the OCNF:starch (1:1 wt%) gels showed an increase in the network strength, as indicated by G′, at lower pH values (Figure 6a). This correlates well with the tan δ data presented in Figure 6b, as the tan δ values decrease with decreasing pH. A similar trend was observed for both the amplitude sweep and viscosity flow curves (Figure S4). However, the control starch dispersions alone showed a decrease in their storage modulus under both acidic and basic pH conditions (Figure 6c). Additionally, the transparency of the starch gels decreased in an acidic medium, as can be seen in Figure 6d. This may be due to the protonation of any negative charge on the starch, leading to charge neutralisation and subsequent aggregate formation, as discussed for the OCNF gel. A decrease in the gel strength of starch at higher pH conditions may be associated with the charge shielding effect of NaOH on the starch chains. Roberts and Cameron [49] suggested that NaOH might influence charge screening of the interaction zones on the starch chains, disrupting the hydrogen bonds responsible for the gel formation. The OCNF:starch blend gels also showed a decrease in their G′ values under higher pH conditions, likely due to the charge shielding effect of NaOH. However, an increase in G′ values of the blend hydrogels under lower pH conditions could be due to the more significant effect of proton-induced interactions (after protonation of the COO^-^) within the cellulose fibrils dominating over the acid-hydrolysed reduction in the molecular size of the starch chains.

**Figure 7:**
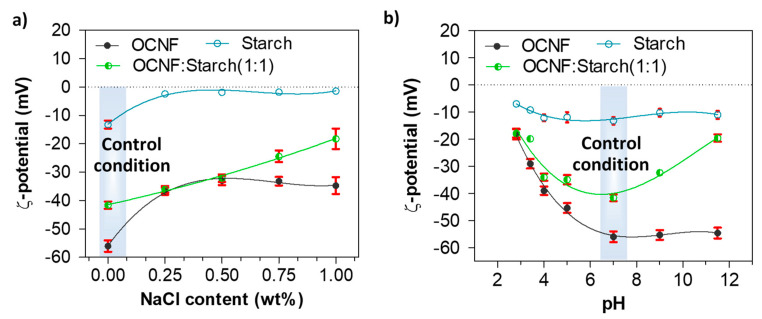
ζ-potential values (with standard deviation, n = 5) of OCNF, starch, and OCNF:starch (1:1) hydrogels (0.1 wt% in DI water): (**a**) with increasing NaCl content up to 1 wt% and (**b**) at various pH conditions.

The role of NaCl and pH on the charge screening of the OCNF:starch hydrogel systems was also investigated via ζ-potential measurements. Figure 7a represents the ζ-potential values of the OCNF:starch gels with varying concentrations of NaCl. The ζ-potential values of the OCNF, starch, and OCNF:starch (1:1 wt%) blend without salt, at pH 7 were found to be −56 (±2), −13 (±1), and −41 (±2) mV, respectively. However, with the addition of salt (up to 0.50 wt%), the magnitude of the ζ-potential values of both OCNF and starch hydrogels decreased. This is due to the adsorption of ions to the OCNF and starch surface, leading to the reduction of the electrical double layer. Thus, a moderate screening of the repulsion forces is expected, triggering particle attraction and forming a stiffer gel network. At a higher salt concentration (>0.50 wt%), the control OCNF and starch-only gels did not show any further decrease in their ζ-potential values, suggesting that 0.50 wt% of salt is the optimum concentration to screen the electrostatic repulsion forces among both the cellulose fibrils and starch chains. This correlates well with the rheology data obtained for these hydrogels. On the other hand, the OCNF-starch blend showed a linear decrease in the magnitude of its ζ-potential with increasing salt content, which could be due to the presence of two components as well as a higher solid fraction in the blend gel. At acidic pH, all the gels also showed a significant reduction in their ζ-potential values due to the protonation on their surfaces. For pH ≥7, the pure OCNF and starch gels did not show any significant difference in their ζ-potential values, while the ζ-potential magnitude was found to decrease for the OCNF:starch blend both at acidic and basic pH (Figure 7b). This seems to contradict results obtained in rheology, where an increase in the gel stiffness was observed only at acidic pH (and assumed to be related to the surface charge screening), while it decreased at basic pH. Here, the effect of NaOH on the blended gels might form a complex, which leads to destabilisation of the gel network with reduced surface charge. This hypothesis is supported by the syneresis observed for these blends at higher pH, a signature of the destabilisation of the system.

**Figure 8:**
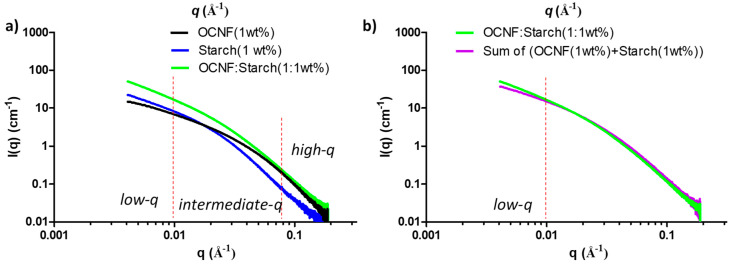
(**a**) Small-angle X-ray scattering (SAXS) patterns of the OCNF:starch blend hydrogels and (**b**) comparison between the SAXS curve of the blend hydrogel (green) with the calculated curve obtained by adding the signal of the two individual components (purple).

SAXS curves of OCNF, starch, and their blend hydrogels are presented in Figure 8a. At low-*q*, the scattering pattern of the OCNF:starch blend hydrogels followed a similar trend as the control starch gels, but with a higher intensity. This indicates that the scattering signal at low-*q* is dominated by the starch. However, in the high-*q* region, the blended gels showed a scattering pattern similar to that of OCNF, which is associated with the fibril cross-section, indicating no significant change in the overall fibril cross-section in the blend hydrogels. A simulated SAXS curve was also obtained by adding the individual contributions of the OCNF (1 wt%) and starch (1 wt%) dispersions (assuming no extra interactions between components) and compared with the measured curve for the OCNF:starch (1:1 wt%) blend hydrogels (see Figure 8b). A clear increase of the slope at the low-*q* region of the blend hydrogels compared to the simulated SAXS curve shows that the measured curve cannot be described as a simple addition of the individual contributions, which suggests the existence of interactions between these two species. Detailed data fitting for the blend hydrogels was not conducted due to the multicomponent nature of the gel system and the fact that the differences in the data were only seen at the low-*q* limit of the recorded data. Proper representation of all components and cross-interactions would inevitably lead to over parametrisation, undermining confidence in the fitting.

**Figure 9:**
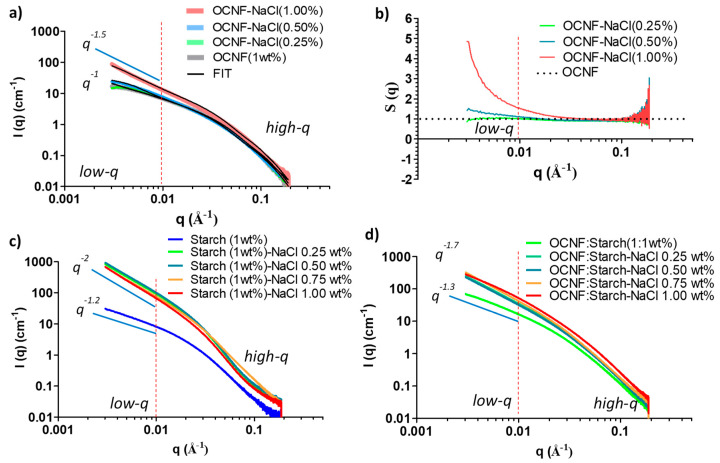
(**a**) SAXS patterns of OCNF (1 wt%) suspensions at various salt concentrations, (**b**) structure factor, *S(q)* of OCNF/salt gels obtained by dividing the signal of the blend gels by the scattering pattern from an OCNF suspension measured without salt, (**c**) SAXS patterns of starch (1 wt%)/salt, and (**d**) the OCNF:starch (1:1 wt%)/salt systems.

The SAXS patterns of the individual OCNF (1 wt%) and starch (1 wt%) suspensions at various NaCl concentrations were also assessed. Patterns taken after the addition of NaCl to the OCNF gels displayed similar high-*q* scattering (see Figure 9a) associated with the OCNF cross-section. In the low-*q* region (0.004 ≤ *q* ≤ 0.01 Å^−1^), scattering from the pure OCNF (1 wt%) gels showed a slope of *q*^−1^, the expected slope at a smaller angle for non-interacting rigid elongated objects like rods or cylinders [35]. The addition of NaCl up to 0.50 wt% to the OCNF gels showed no clear increase in the intensity or change in the slope in the low-*q* region, suggesting the stability of the salt-induced gel network formed. Hence, no precipitation was observed in the case of OCNF gels up to 0.50 wt% NaCl concentration, however, these suspensions had increased gel strength.

The SAXS data obtained for the control OCNF and 0.25 wt% NaCl content OCNF gels were fitted using a rigid elliptical cylindrical model [35] (see Figure 9a for the fits and Table S1 for the fitting parameters), which is in good agreement with the previous reports suggested by Schmitt et al. [35]. However, in the case of the higher salt content gels (0.50 wt% and more), the SAXS data were fitted using a semi-flexible elliptical cylinder model [35], indicating a physical change in the arrangement of fibrils due to the appearance of points of contact between fibrils. This is suggested to be due to the addition of counter ions on the cellulose surface. It is to be noted that the flexibility parameters of the model used by Schmitt et al. are a reflection of the overall mesh size rather than the flexibility of the fibrils themselves and could also be purely phenomenological [35], as an easy way to pinpoint a change in the fibril–fibril attraction.

SAXS patterns for OCNF gels with higher salt concentration (1 wt%) showed an apparent increase in the scattered intensity as well as the change of slope (to *q*^−1.5^) in the low-*q* region, suggesting increased fibril–fibril attraction, even perhaps fibril–fibril aggregation. This is in agreement with the comparatively lower LVE region, as observed in the amplitude sweep curve for this gel system. To show this attraction, the change in structure factor [S(*q*)] with the addition of salt was also calculated. Results are presented in Figure 9b. The addition of 0.25 wt% NaCl to the OCNF (1 wt%) gels did not reveal any change in their S(*q*) compared to the pure OCNF, while 0.50 wt% of NaCl in the OCNF gels showed a clear divergence [S(*q*) > 1] in the low-*q* region, suggesting the presence of salt-induced attractive interactions among the fibrils. A considerable divergence of S(*q*) value for OCNF gels with 1 wt% NaCl was observed, which further points toward the formation of larger aggregates of these negatively charged fibrils due to stronger fibril attraction at higher ionic strengths.

The SAXS patterns of starch at various salt concentrations were also investigated, as presented in Figure 9c. A significant increase in the scattered intensities as well as the slope of the data (from *q*^−1.2^ to *q*^−2^ at low-*q*) clearly demonstrates the salt-induced interactions among the starch molecules. Moreover, the slope obtained for starch with salt (*q*^−2^) was even higher than that of OCNF with salt (*q*^−1.5^), suggesting a more pronounced level of interactions of Na^+^ and Cl^−^ ions with the starch molecules, inducing stronger attractions among them, or even aggregation into a fractal network or sheet-like structures [50]. This correlates with the visible increase in the turbidity of the gels, as shown earlier in Figure 3b and also in Figure S2.

Patterns taken from samples after the addition of salt to the OCNF:starch blend hydrogels showed an increase in their scattered intensity, more evident in the low-*q* region (Figure 9d), suggesting long-range salt-induced interactions among the cellulose fibrils and starch molecules. However, the presence of salt was also suggested to compete with the starch for the bound water, resulting in slower retrogradation [44,45,46,47]. Hence, despite having a greater interaction with salt, no gels were formed in the case of the starch-salt system (Figure S2).

**Figure 10:**
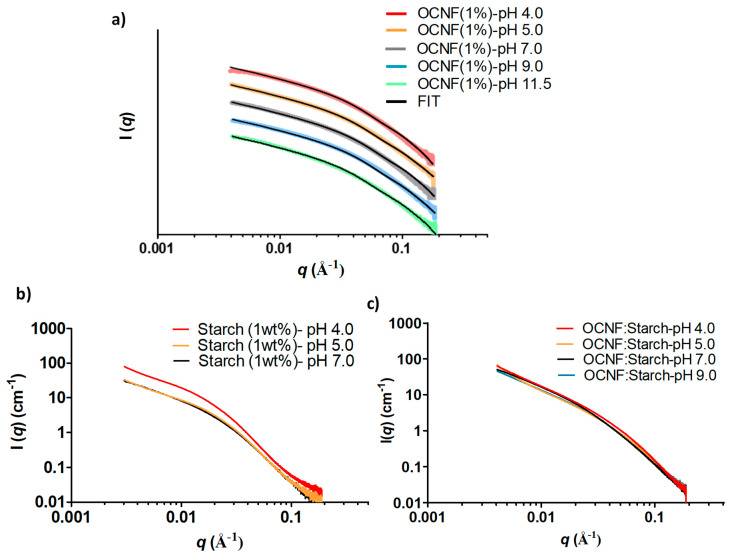
SAXS patterns of the gels at various pH: (**a**) OCNF (1 wt%) (offset was used for clarity), (**b**) starch (1 wt%), and (**c**) OCNF:starch (1:1 wt%).

At various pH conditions, the SAXS patterns of OCNF (1 wt%) gels did not show any pronounced difference, except for the scattering pattern of the OCNF gel obtained at pH 4.0 at low-*q* (Figure 10a). The divergence of this scattering signal from the fitted data (rigid elliptical cylinder model) at low-*q* is likely to be associated with proton-induced aggregated fibrillar network formation, which is also suggested by the increase in storage modulus obtained for this gel. On the other hand, starch gels at pH 5.0 and 7.0 showed similar scattering patterns (see Figure 10b), while at pH 4.0, the intensity of the scattering pattern was found to be higher due to proton-induced interactions causing the formation of scattering domains with increased electron density. Similar scattering patterns were also observed for the OCNF:starch gels at pH 4.0, 5.0, 7.0, and 9.0. The scattering patterns of starch (at pH 9.0 and 11.5) and OCNF:starch (at pH 11.5) were not recorded as syneresis was observed in the capillary during the SAXS measurements, indicating the collapse of the gel network in these samples.

## 4. Conclusions

In this study, NaCl and pH-induced gelation of OCNF and starch in water systems were investigated. A progressive increase in the storage modulus of OCNF-only hydrogels with increasing salt concentration demonstrated the increase in the stiffness of the gels due to the surface charge screening of the cellulose fibrils by the counter ions. Additionally, the change in repulsive interactions (ζ-potential ~ −56 mV) to the attractive regime (zeta potential around −30 mV or lower) corresponded to their low-frequency dependent G′ and tanδ = G′’/G′ < 1 values in rheology, suggesting physical gel-like behaviour. When starch is added, the stiffness and viscosity of OCNF:starch hydrogels were further enhanced by the addition of NaCl (up to 0.5 wt%, ~86 mM). Increased ionic strength within the gel network disrupts the double layer of the charged particles, allowing stronger interactions between cellulose fibrils and starch molecules. However, at higher ionic strength (i.e., 0.75 wt% NaCl content or more), the OCNF:starch gel strength appears to reduce due to the formation of larger aggregated gel networks (resulting in precipitation). The OCNF:starch hydrogels were found to be stable in a pH range from 5.0 to 9.0, although at lower pH (=4.0), proton-induced fibrillar interaction was suggested to form stiffer gels. The reduced magnitude of the ζ-potential values obtained after the addition of salt as well as lowering the pH level was suggested to increase these interactions, promoting the aggregation behaviour of these hydrogels. SAXS analysis of OCNF also suggested that adding salt increased attractive interactions as observed by the increase in the slope at the low-*q* region arising from larger structures forming due to the screening of the surface charge of the fibrils. This work provides an in-depth understanding of the OCNF:starch gel network, which has the potential to be utilised as a rheological modifier in food, personal, and health care products.

## Data Availability

Data supporting this work are freely accessible in the Bath research data archive system at https://doi.org/10.15125/BATH-00985.

## Supplementary Materials

The following are available online at https://www.mdpi.com/2073-4360/13/6/951/s1, Figure S1: (a) Elution profiles of pullulan standards and (b) standard curve obtained from the elution profile of pullulan standards. Figure S2: (a) Frequency sweep curves and (b) photographs of the starch (1 wt%)/NaCl (0, 0.25, 0.5, 0.75, or 1 wt%) hydrogels. Figure S3: Photographs show the phase separation of OCNF (1 wt%) hydrogels after the rheological test produced at pH 3.4 (top) and no phase separation for the gels produced at pH 4.0 (bottom). Here, a 80 mm glass plate was used to visualise the gels’ physical properties during rheological tests. Figure S4: (a) amplitude sweeps, and (b) shear flow curves of OCNF:starch (1:1 wt%) hydrogels made at various pH conditions (4, 5, 7, 9, 11.5). Table S1: The parameters obtained by fitting of the SAXS data via rigid and flexible elliptical cylinder models. The length of the OCNF was fixed at 1600 Å based on the average length obtained via the TEM image analysis, except for the OCNF(1%)-NaCl(1%) gel, where a longer length (4000 Å) was used to fit the experimental data. This apparent increase in length might be associated with the salt-induced (at higher concentration, 1%) longitudinally assembled fibres.
