# Supplementary material for: Monovalent Salt and pH-Induced Gelation of Oxidised Cellulose Nanofibrils and Starch Networks: Combining Rheology and Small-Angle X-ray Scattering
Source: Polymers (Basel). 2021 Mar 19;13(6):951. doi: 10.3390/polym13060951 (PMC8003611; doi:10.3390/polym13060951)
Supplement: Supplementary file 1 [file polymers-13-00951-s001.pdf]

Electronic supplementary information (ESI)

Monovalent salt and pH-induced gelation of oxidised cellulose nanofibrils and starch networks: combining rheology and small-angle X-ray scattering

Kazi M. Zakir Hossain[a], Vincenzo Calabrese[a], Marcelo A. da Silva[a], Saffron J. Bryant[a], Julien Schmitt[a†], Jennifer H. Ahn-Jarvis[b], Frederick J. Warren[b], Yaroslav Z. Khimyak[c], Janet L. Scott[ad], and Karen J. Edler[ad*]

[a]Department of Chemistry, University of Bath, Claverton Down, Bath, BA2 7AY, UK

[b]Food Innovation and Health, Quadram Institute Bioscience, Norwich Research Park, Norwich, UK, NR4 7UQ

[c]School of Pharmacy, University of East Anglia, Norwich NR4 7TJ, UK

[d]Centre for Sustainable Chemical Technologies, University of Bath, Claverton Down, Bath, BA2 7AY, UK

[†]current address: LSFC - Laboratoire de Synthèse et Fonctionnalisation des Céramiques, UMR 3080 CNRS / Saint-Gobain CREE, Saint-Gobain Research Provence, 550 avenue Alphonse Jauffret, Cavaillon, France

[*]Corresponding author: K.Edler@bath.ac.uk

a)

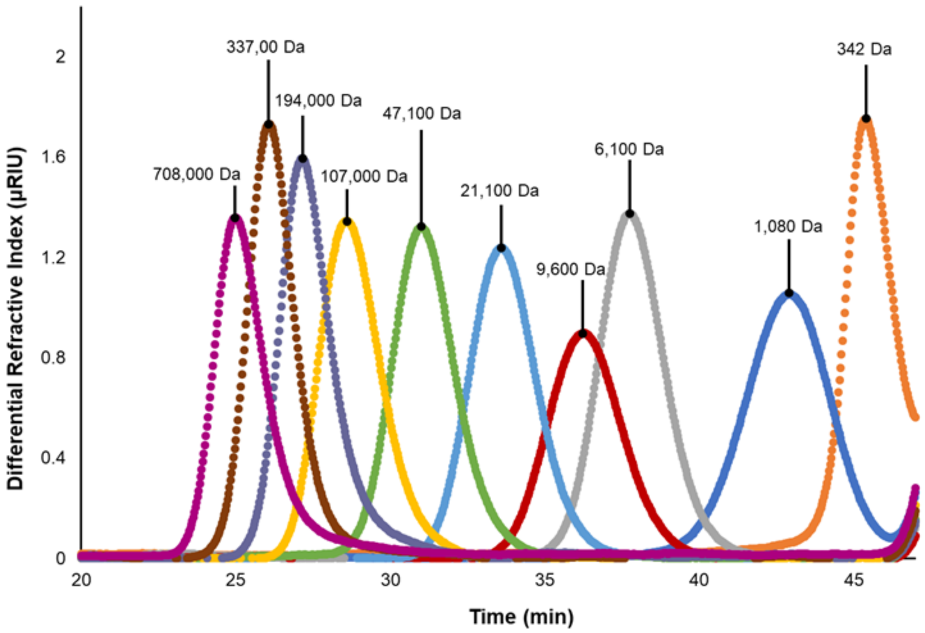

b)

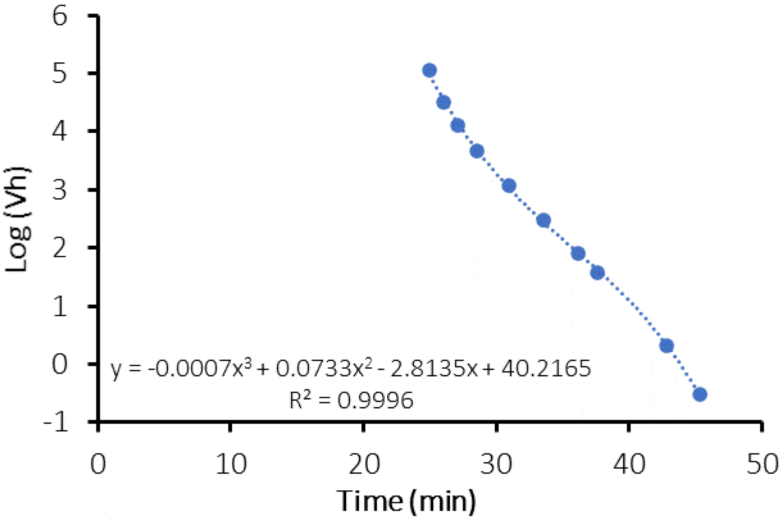

Figure S1: a) Elution profiles of pullulan standards and **b)** standard curve obtained from the elution profile of pullulan standards.

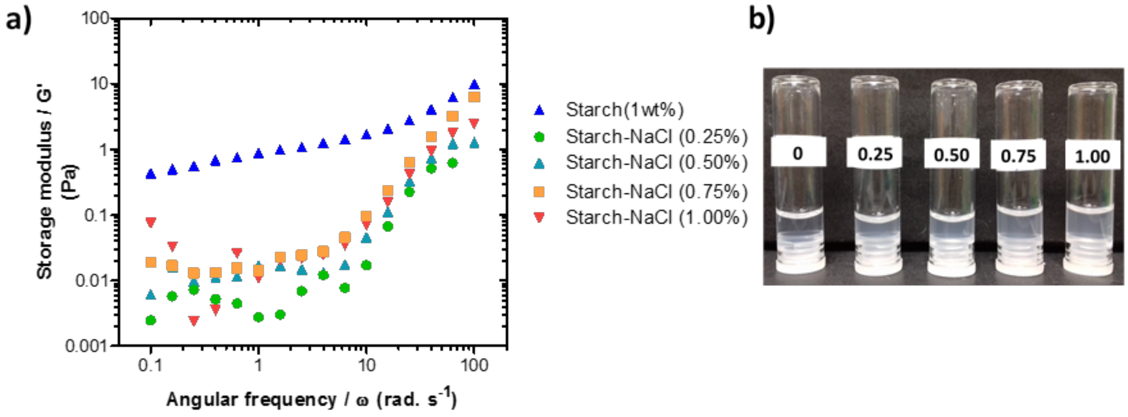

Figure S2: a) Frequency sweep curves and b) photographs of the starch (1 wt%)/NaCl (0, 0.25, 0.5, 0.75 or 1 wt%) hydrogels.

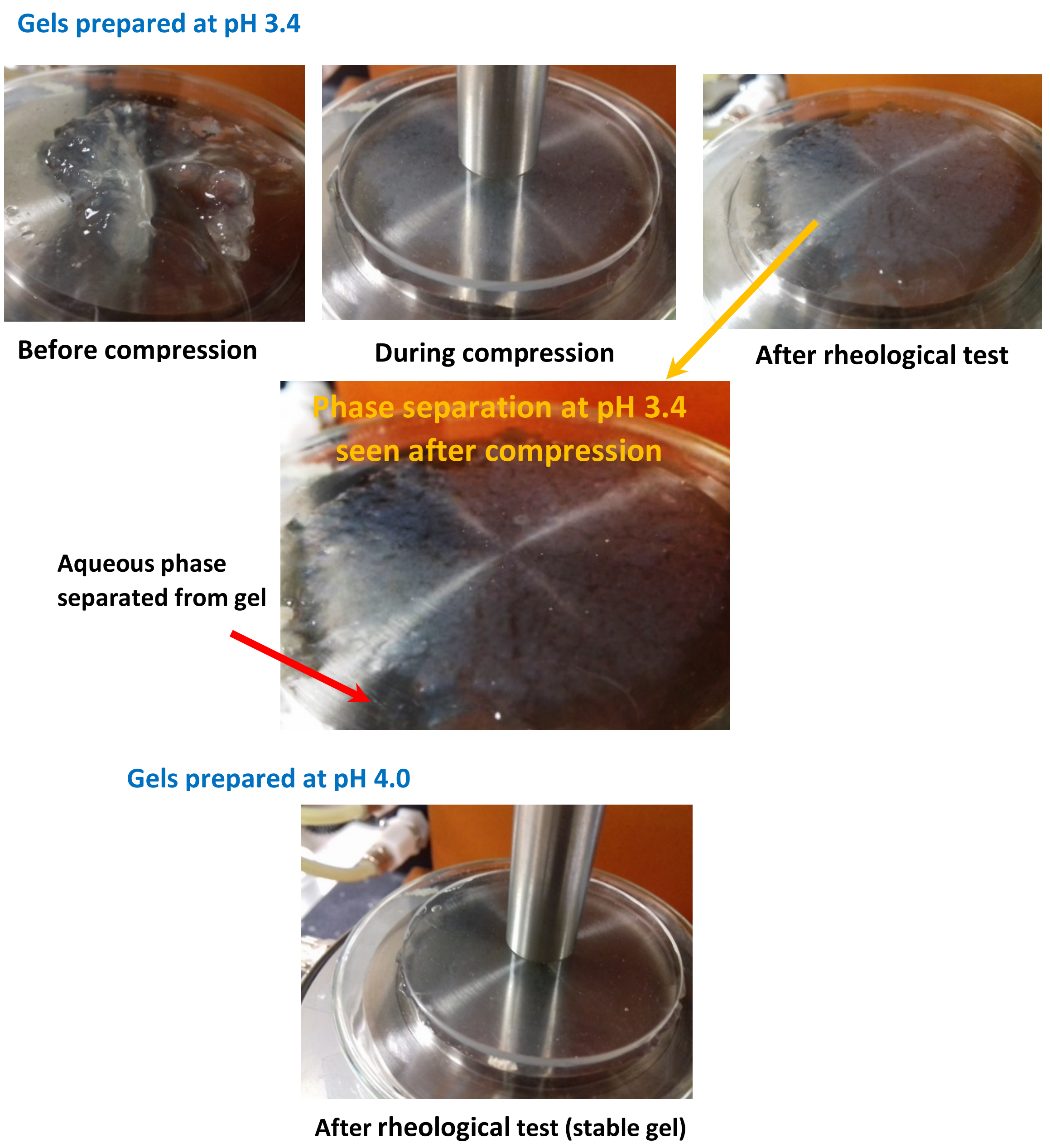

Figure S3: Photographs show the phase separation of OCNF (1 wt%) hydrogels after rheological test produced at pH 3.4 (top) and no phase separation for the gels produced at pH 4.0 (bottom). Here, a 80 mm glass plate was used to visualise the gels physical properties during rheological tests.

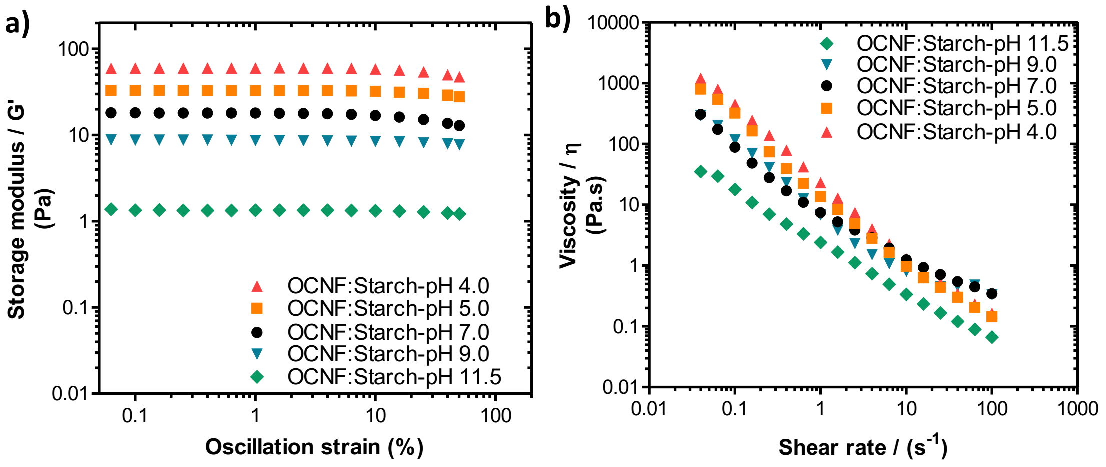

Figure S4: a) amplitude sweeps, and b) shear flow curves of OCNF:Starch (1:1 wt%) hydrogels made at various pH conditions (4, 5, 7, 9, 11.5).

Table S1: The parameters obtained by fitting of SAXS data via rigid and flexible elliptical cylinder models. The length of the OCNF was fixed at 1600 Å based on the average length obtained via the TEM image analysis,[26] except for the OCNF(1%)-NaCl(1%) gel, where a longer length (4000 Å) was used to fit the experimental data. This apparent increase in length might be associated with the salt-induced (at higher concentration, 1%) longitudinally assembled fibres.

SLD (scattering length density) for cellulose: 13.5 x10^{-6} Å^{-2}

SLD solvent: 9.5 x10^{-6} Å^{-2}

	Model	Radius-minor (Å)	Axis ratio	Kuhn length (Å)	Length (Å)
OCNF(1%)	Rigid elliptical cylinder	11±2	4.8	-	1600
OCNF(1%)-0.25% NaCl	Rigid elliptical cylinder	11.5±2	5.2	-	1600
OCNF(1%)-0.50% NaCl	Flexible elliptical cylinder	11.8±2	5.0	600	1600
OCNF(1%)-1.00% NaCl	Flexible elliptical cylinder	12.5±2	5.0	400	4000
OCNF(1%)-pH 4.0	Rigid elliptical cylinder	11.5±2	4.8	-	1600
OCNF(1%)-pH 5.0	Rigid elliptical cylinder	11.5±2	4.8	-	1600
OCNF(1%)-pH 9.0	Rigid elliptical cylinder	11.5±2	4.5	-	1600
OCNF(1%)-pH 11.5	Rigid elliptical cylinder	12.5±2	4.5	-	1600
